# A Discrete Electromechanical Model for Human Cardiac Tissue: Effects of Stretch-Activated Currents and Stretch Conditions on Restitution Properties and Spiral Wave Dynamics

**DOI:** 10.1371/journal.pone.0059317

**Published:** 2013-03-19

**Authors:** Louis D. Weise, Alexander V. Panfilov

**Affiliations:** 1 Department of Theoretical Biology, Utrecht University, Utrecht, The Netherlands; 2 Department of Physics and Astronomy, Ghent University, Ghent, Belgium; Universitat Politecnica de Catalunya, Spain

## Abstract

We introduce an electromechanical model for human cardiac tissue which couples a biophysical model of cardiac excitation (Tusscher, Noble, Noble, Panfilov, 2006) and tension development (adjusted Niederer, Hunter, Smith, 2006 model) with a discrete elastic mass-lattice model. The equations for the excitation processes are solved with a finite difference approach, and the equations of the mass-lattice model are solved using Verlet integration. This allows the coupled problem to be solved with high numerical resolution. Passive mechanical properties of the mass-lattice model are described by a generalized Hooke's law for finite deformations (Seth material). Active mechanical contraction is initiated by changes of the intracellular calcium concentration, which is a variable of the electrical model. Mechanical deformation feeds back on the electrophysiology via stretch-activated ion channels whose conductivity is controlled by the local stretch of the medium. We apply the model to study how stretch-activated currents affect the action potential shape, restitution properties, and dynamics of spiral waves, under constant stretch, and dynamic stretch caused by active mechanical contraction. We find that stretch conditions substantially affect these properties via stretch-activated currents. In constantly stretched medium, we observe a substantial decrease in conduction velocity, and an increase of action potential duration; whereas, with dynamic stretch, action potential duration is increased only slightly, and the conduction velocity restitution curve becomes biphasic. Moreover, in constantly stretched medium, we find an increase of the core size and period of a spiral wave, but no change in rotation dynamics; in contrast, in the dynamically stretching medium, we observe spiral drift. Our results may be important to understand how altered stretch conditions affect the heart's functioning.

## Introduction

The heartbeat is governed by electrical waves of excitation that periodically propagate through the cardiac muscle and initiate its contraction. Abnormal electrical excitation of the heart may result in cardiac arrhythmias disturbing the heart's pumping function. Heart failure due to cardiac arrhythmias is a major cause of death in the industrialized world [1]. It is known that dangerous types of arrhythmias are caused by spiral waves of electrical excitation in the cardiac muscle [2]–[4].

Electrical waves of excitation are affected by the deformation of the heart via the mechano-electrical feedback phenomenon. It has been shown that the rapid stretching of cardiac tissue (mechanical stimulation) has a significant effect on the heart's functioning, for example, due to the initiation of electrical waves [5], [6]. Important examples are “commotio cordis” [7], [8], the phenomenon that an impact on the chest can cause arrhythmia; and the “precordial thump”, the phenomenon that an impact on the chest of a patient may stop an arrhythmic heart condition [9]. Both phenomena are believed to be a result of an abrupt deformation of the heart, and the main effect of deformation on the electrical activity is considered to be transmitted via so-called stretch-activated ion channels. These channels produce depolarizing inward current as a response to stretch of the tissue [5]. The study of mechano-electrical feedback is an important direction of research in current cardiac electrophysiology [10].

A valuable method to study mechano-electrical feedback is mathematical modeling allowing to study the coupled mechanical and electrical activity of the heart, which is a difficult problem in experimental research. Generic electromechanical models for heart tissue are successfully applied, for instance, to investigate the effect of mechano-electrical feedback on pacemaking and spiral wave activity [11]–[15] and to find mechanisms for the onset of spiral waves [16], [17]. Yet, these generic models are limited to studies on a qualitative level, and more detailed models for cardiac tissue need to be developed. First steps in that direction have been made by coupling continuous mechanical models to biophysical models of cardiac excitation and contraction [18], [19]. However, continuum mechanics is computationally demanding and makes it difficult to achieve high spatial and temporal resolution of the coupled electrical and mechanical processes. In this paper, we introduce an electromechanical model for human cardiac tissue which couples detailed biophysical models for cardiac excitation and contraction to a discrete mechanical model. We use an ionic model of excitation for human cardiac cells (Tusscher, Noble, Noble, Panfilov 2006 model) [20], [21] and a biophysical model for excitation-contraction coupling (adjusted Niederer, Hunter, Smith, 2006 model) [22], [23]. Our method applies a generic model for cardiac elasticity, an ideal crystal lattice of mass points connected with springs. The mass-lattice model describes a material which was introduced by Seth 1935 to discuss problems of finite strain [24]. The Seth material relation is an extension of the generalized Hooke's law to finite elasticity. To solve the mechanical equations we apply the Verlet integration [25] (explicit, finite difference backwards integration scheme), a method which is widely used in molecular dynamics simulations. The Verlet integration has first been used to solve mass-lattice models in cardiac elasticity by Mohr [26]. We applied this discrete mechanical description before in a model to study reaction-diffusion-mechanics systems [15], and use it here to set up an electromechanical model for cardiac tissue. An advantage of this method is its computational efficiency which allows to solve the coupled electromechanical equations with high spatiotemporal resolution.

To demonstrate the value of the discrete electromechanical model we apply it to study effects of deformation on basic properties of cardiac tissue, such as the action potential shape, restitution properties, and the dynamics of spiral waves. In this study we consider two mechanical conditions. The first one is a constantly stretched medium, a simple assumption which was widely used in initial studies on mechano-electric feedback [27], [28], and mimics conditions such as dilated cardiomyopathies [27]. As a second condition, we assume a deforming medium which resembles deformation occurring as a result of cardiac contraction. We find that these stretch conditions have very different effects on the studied characteristics of excitation, and discuss the underlying mechanisms. The results of our application study may be important to understand how situations of increased mechanical load in the cardiac muscle alter the qualitative effect of stretch-activated currents.

## Methods

### Model for Cardiac Excitation

We use the 2006 version of the Tusscher Noble Noble Panfilov model for human epicardial myocytes (TP06) [20], [21]. The model is given as the following reaction-diffusion equation for the transmembrane potential 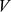


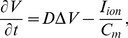
(1)with the membrane capacitance density 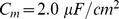, the diffusivity 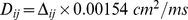, and the transmembrane ion current
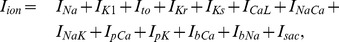
(2)where 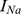 is fast Na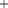 current, 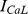 is L-type Ca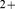 current, and the K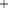 currents are 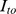 (transient outward), 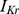 (rapid delayed rectifier), 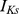 (slow delayed rectifier), and 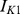 (inward rectifier). Furthermore, 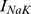 is the Na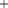/K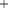 pump current, 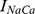 is the Na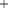/Ca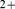 exchanger current, 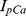, 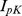 are plateau Ca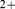 and K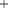 currents, and 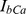, 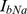 are background Ca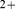 and Na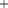 currents. The voltage dependency of ion channels is modeled [29] by gating variables with dynamics of the form
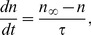
(3)where 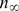 describes the voltage-dependent steady state activation, and 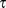 the voltage-dependent characteristic time for a respective gating variable. The TP06 model also describes Ca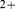 dynamics of intracellular compartments of the sarcoplasmatic reticulum. A list of parameters and equations for these currents is given in [20].

In our model we add a stretch-activated depolarizing current 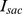 which will be introduced in the section “Mechano-Electrical Feedback”.

We will now describe the coupling of the electrical excitation process of the cardiomyocytes to their tension development.

### Model for Excitation-Contraction Coupling

We model myocyte excitation-contraction coupling in our model with a numerically improved version of the Niederer, Hunter, Smith (NHS) model [22], [23] adjusted to human cardiac tissue. The NHS model describes active tension in a sarcomere as a function of intracellular calcium concentration [Ca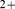]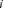, sarcomere length, and the rate of sarcomere length change, determinants which have been shown to substantially affect the active tension development (see [22] and references within).

The NHS model takes the dynamics of sarcomere length into account. We follow previous studies [12], [15]–[17] and define as a pseudo normalized sarcomere length.
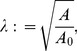
(4)where the 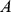 is the surface area of a smallest area element in the model (see section “Mass-Lattice Model”), and 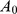 is the surface area of such a smallest area element in undeformed state.

#### Adjustments for Human Ventricular Cells

We followed changes on the original version of the NHS model [22], which was originally set up using experimental data of rat and guinea pig hearts, that have been made in the work [18] to model human ventricular myocytes. These changes were explained in [18] by experimentally observed relaxation rates due to higher body temperatures [30]. The changes are a speeding up of myocyte relaxation rates to 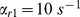, 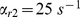, and adjusting the contractile tension by setting parameters T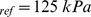 (maximum contractile tension at resting length of sarcomere) and pCa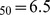 (p[Ca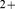]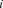 at half maximal contractile tension).

#### Active Tension in Myocytes

The NHS model describes the tension 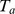 development in cross bridges as.
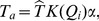
(5)

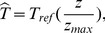
(6)where 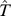 is the length and velocity independent tension, (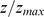) is the fraction of available actin sites in a sarcomere 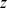 to the maximal available actin sites 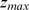 at a particular sarcomere length. Variable 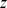 provides the coupling of the electrical and mechanical system and is found during integration of the NHS model where it is directly related to [Ca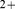]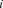, which is given by the electrical equations of the TP06 model. Scaling functions 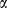 and 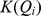 describe the sarcomere length and velocity dependencies of the total tension [22]. In particular, the function 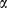 models the influence of the dynamics of the cross bridge cycle and thin filaments in a sarcomere, and function 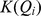 accounts for the velocity dependency of tension development via a fading memory model.

It has been shown that in strongly coupled electromechanical models for cardiac tissue, in which the equations for excitation processes and mechanical processes are jointly solved, computational difficulties may occur due to the velocity and length-dependency of a sarcomere [23]. To solve this problem, Niederer and Smith proposed the “update method” [23], where the functions 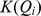 and 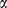 are continuously calculated within the mechanical iteration algorithm to calculate the total tension 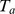 via Eq.(5). In this paper, we apply the NHS model, adjusted for human cardiac tissue, together with the “update method”. For a detailed model description and parameters, see [22], [23].

We will now describe the passive elastic properties of the medium.

### Mass-Lattice Model

**Figure 1:**
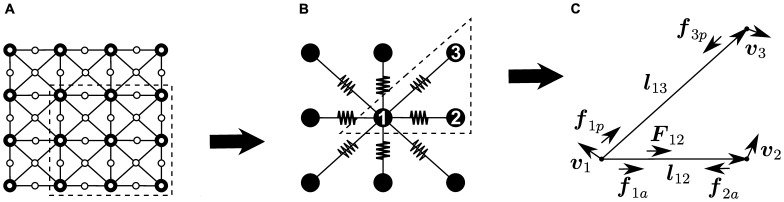
Coupled mechanical and electrical mesh. (**A**) Coupled mechanical and electrical mesh. The mass points are indicated as large black dots. The finite difference points to solve Eq.(1) are indicated as small white dots. The lattice springs are indicated as black lines. (**B**) Unit cell of the two-dimensional lattice. Mass point **1** and its four horizontal and vertical nearest neighbors and four diagonal next-nearest neighbors are connected with direct active and diagonal passive springs. Lattice springs are indicated by zigzagging lines (fat lines for active and thin lines for passive springs). Dotted contours indicate insets for the associated subfigures. (**C**) Vectors used in Eqs.(7)-(10) for calculating lattice interactions. Figure taken from [15].

We use the mass-lattice framework introduced in [15]. The two-dimensional lattice consists of material points connected by springs (Figure 1A). In this square lattice each mass point is connected to 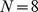 (if not at the boundary) direct neighboring mass points with springs that follow Hooke's law (Figure 1B). The equations of the model are.
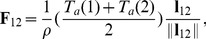
(7)

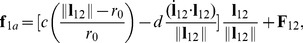
(8)

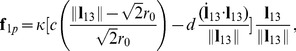
(9)

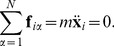
(10)


Figure 1C illustrates main forces and the displacements of active and passive lattice springs connecting the mass point **1** to the mass points **2** and **3**. Each mass point is connected to its 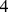 diagonal neighbors with “passive springs” (passive elastic properties), and to its 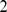 vertical and 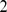 horizontal neighbors with “active springs” (passive and active forces). Eq.(7) describes the excitation-contraction coupling of two neighboring mass points 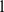 and 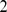 connected with an active spring, where 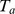 is active tension from Eq.(5), and 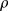 is the mass point surface density (see section “Numerical Methods”). Eqs.(8),(9) describe forces 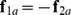 mediated through an active spring to mass points 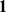 and 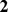, and forces 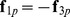 mediated through a passive spring to mass points 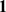 and 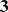. In Eqs.(8),(9) the spring vectors are given by mass point's positions as 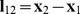 and 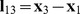, 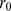 is the resting length of an active spring and 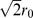 the resting length of a passive spring, 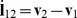 and 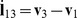 are the time derivatives of the respective spring vectors 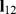, 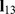. Parameter 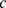 is the stiffness constant, and 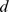 is the damping parameter. Parameter 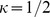 is the stiffness ratio between active and passive springs which causes the lattice to be macroscopically isotropic [31] for small deformations, and can be described by the generalized Hooke's law.
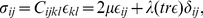
(11)with the small strain tensor 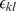, Cauchy's stress tensor 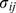, linear elasticity tensor 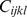, Kronecker delta 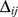, and Lamé coefficients 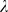 and 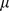. Krivtsov showed in [32] that the lattice can be approximated by the Seth material relation [24] for non-linear deformations which is given by Eq.(11) when the Almansi's finite strain tensor is used instead of the small strain tensor. Young's elastic modulus of cardiac tissue has been measured in an atomic force microscopy study 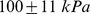 [33]. However, cardiac tissue provides a nonlinear elastic behavior for larger deformations, and we found that setting Young's elastic modulus to 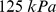 in our model results in maximal deformations of springs of 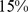, similar to contracting cardiac cells. Thus we set the spring stiffness 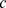 accordingly to 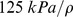 (see section “Numerical Methods”). As we assume elastostatics 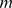 and 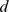 have no physical relevance and should be set to optimize computations. Following [15], we set 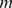 to the identical numerical value of 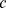 (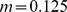 for 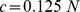 compare section “Numerical Methods”), and set 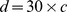 to achieve stable and efficient computations (see section “Model Validation”).

In section “Model for Excitation-Contraction Coupling” we defined a pseudo normalized sarcomere length 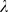 in terms of the surface area of a smallest area element 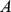 in the lattice (see Eq.(4)), which is a quadrilateral formed by 4 direct neighboring mass points connected with active springs (see Figure 1A). Parameter 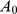 is the surface area of such a smallest area element in the undeformed model, 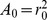 (see Figure 1A).

We will now describe how we model mechano-electrical feedback via stretch-activated currents.

### Mechano-Electrical Feedback

The deformation of a cardiomyocyte affects its excitation processes. It has been shown in studies of excised cardiac tissue and the whole heart that a direct electrophysiological influence of the deformation of cardiac tissue is a depolarizing stretch-activated current 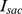 (compare Eq.(2)) through stretch-activated ion channels [5]. Experimental studies have shown that these channels are activated instantaneously with mechanical stretch and follow a linear current-voltage relationship [34], [35]. Linear, time-independent models have been proposed for 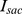 [27], [28], and have been used in other electromechanical models [12], [14], [18]. Following these previous studies we use.
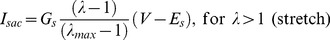
(12)where 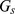 is the maximal conductance, and 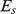 is the reversal potential of the stretch activated channels. For 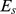 values around 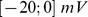 have been reported [36], [37], and we set 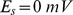. It has been shown that 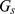 is within 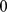 to 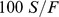 [5], [38], and in this paper we vary 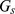 in this range to study the influence of 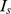 on several properties. Parameter 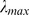 is the maximal pseudo normalized sarcomere length which we set to 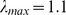 as in [18].

### Numerical Methods

We solved the model applying an explicit Euler method for the TP06 and NHS models, and Verlet integration [25] for the mechanical model. After each Euler computation of the electrical system a new [Ca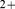]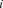 is obtained, and a length and velocity independent tension 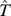 is computed via Eq.(6). 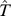 is then passed to the mechanics model, where the mechanical equations are solved, using Verlet integration time step 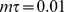, until the sum of forces on each mass point is smaller than threshold 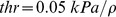. The Verlet computation of the position of a mass point 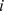 for integration time 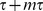 is
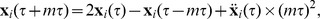
where 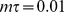 is the Verlet integration time step and 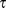 is the integration time. For the very first time step, we use



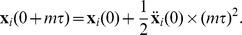



The acceleration of a mass point 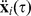 is given by Eq.10. At each time step the velocities of the mass points are calculated by
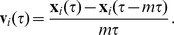



During the mechanical iteration algorithm the length and velocity dependent tension scaling functions 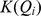 and 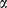 of the total tension 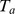 which is computed via Eq.(5) are updated together with the mesh configuration using the “update-method” [23]. We found that numerical difficulties can occur in situations when the damping force in a spring exceeds the Hooke's force, for example in an active spring (see section “Mass-Lattice Model”).
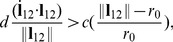
(13)which in turn causes slow convergence of the iterative algorithm requiring in some cases thousands of iterations before convergence. However, we found that good convergence can be achieved by setting the absolute damping force to 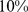 of the Hooke's spring force (for springs for which condition of Eq.(13) is true). With this, we observe a significant improvement - typically the mechanical system converges within 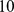
–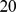 iterations. In this paper the “update method” is applied within the Verlet routine on discrete nodes, whereas in the original work [23] the method is used within the Newton algorithm to solve equations of continuum mechanics. Moreover, here the actual sarcomere length was not used for the mechano-electrical feedback calculation, but a pseudo normalized sarcomere length 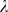 from Eq.(4). For simulations we used an Euler integration space step from 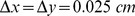 to 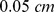 and Euler integration time step of 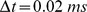. We computed a quadratic grid of up to 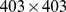 finite difference points (and up to 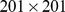 mass points) using no-flux boundary conditions modeling a thin quadratic layer of 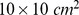 cardiac tissue. The spring stiffness 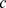 and mechanical threshold 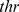 are functions of the mass point density. Mass point density 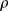 is function of the coarseness of the mass-lattice model, here we set the resting length of an active spring to be as long as two finite difference integration steps 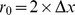 (see Figure 1A), thus the mass point density is



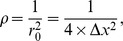
where the factor 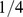 is the ratio of mesh coarseness (#mechanical points/#electrical points) in the model. For an Euler space step of 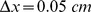, the mass point density is 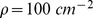, spring stiffness is 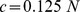, and the mechanical threshold is 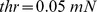. The boundaries of the medium were fixed in space modeling isometric contraction to mimic isovolumic phases in the cardiac cycle, a common assumption which was used in similar electromechanical studies [12], [14]–[16].

### Model Validation

The numerical coupling and integration of the Euler and the Verlet scheme require the choice of several parameters. We will first discuss the integration parameters and then parameters for the coupling of the numerical grids to assure efficient and stable computations.

#### Integration Parameters

It has been shown that the TP06 model together with the improved NHS model coupled with a whole heart continuum mechanics model can be stably integrated with the Euler method using integration parameters 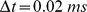 and 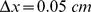 [18]. We use Euler integration steps of 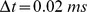 and 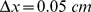 as in [15] for most computations, and use a stricter setting 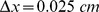 for simulations on spiral wave dynamics. To validate the usage of larger space step 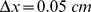 we performed simulations of our main results on potential shape, and restitution properties using 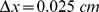. In these simulations we found that our setting of Euler integration parameters yields consistent results. We update the mechanical configuration after each Euler step, and achieve stable and accurate integration of the coupled electromechanical model. For solving the mechanical Eqs.(8)-(10) we use a Verlet integration time step of 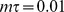 (as in [15]). We find that this setting allows efficient and stable computations of new configurations of the mechanical grid for this paper's simulations.

#### Damping-Stiffness-Ratio

The system of coupled, damped, driven, mechanical oscillators described by Eqs.(8)-(10) has been shown in [15] to allow fast stable convergence of the lattice mass points to their new configuration in a similar application as in this paper. In this work we found that setting the damping-stiffness ratio 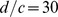 (dimensionless) as in [15] yields stable and efficient computations of mechanical mesh configurations in most situations. However, we found that in some situations (e.g. under external stimulation) we get numerical difficulties when Eq.(13) is true even for smaller values of the damping-stiffness ratio 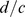 (e.g. 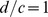). Thus, we apply 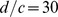 together with the stability criterion outlined in the section “Numerical Methods” for all this paper's simulations.

#### Electrical and Mechanical Grids

In [15] we applied a method to validate the mesh coupling of the finite difference mesh and the mechanical mesh via an error norm defined by residues of mass point trajectories. This method allowed us to find accurate coupling parameters. We found in these validation experiments that the usage of a coarser finite difference mesh compared to the mechanical mesh as shown in Figure 1A allows accurate computations. This is, because changes in tension and strain are typically distributed more smoothly in space than electrical variables. For example, the upstroke of an electrical excitation wave has a length of the order of one to two millimeters, whereas the mechanical tension changes over a range of few centimeters. In this paper, we performed simulations to test how a change in resolution of the mechanical mesh compared to the electrical system affects the main results of the paper, and we found that our parameter setting yields consistent results. We also found in [15] that our mechanics model converges better with a frequent update rate, and therefore we also choose here to update the mechanical mesh after each time the electrical system was solved (every 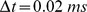). We performed a convergence study to determine a suitable value for the threshold of convergence for the mechanical problem for numerical step sizes. For this we halved and quartered the value for 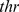, and found qualitatively same results (influence of stretch on action potential, restitution properties, spiral wave dynamics). Thus we set 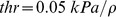, e.g. for 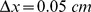, 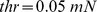, and for 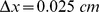, 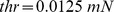.

#### Mesh Initial Conditions

**Figure 2:**
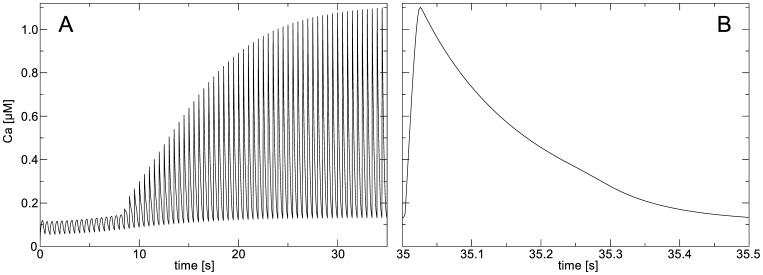
Mesh initialization (A) [Ca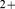 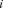] transient during pacing experiment. [Ca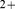]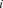 is shown for a single non-deforming cell undergoing 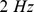 pacing. (**B**) Steady state [Ca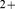 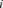 **]** transient. [Ca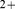]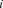 is shown for a single non-deforming cell after 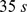 of 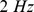 pacing.

The main determinant of cardiac contraction is the [Ca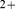]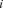 transient, and it is necessary to set initial conditions of the TP06 model so that it describes a steady state Ca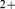 dynamics of a working cardiac cell. We found that one should carefully approach this problem as establishing of such a steady state can take a substantial period of time. To demonstrate this, we performed a numerical experiment on a single, non-deforming cell in which we stimulated it with a frequency of 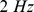 by setting 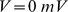 for one time step 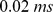. We see in Figure 2A that it requires a long time to reach steady state dynamics for [Ca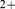]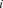. Figure 2B illustrates the [Ca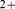]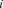 transient after 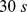 of applying the stimulation protocol. According to this simulation we adjusted initial conditions of the TP06 model: [Ca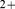]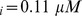; [CaSR]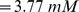; CaSS 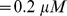. Note that these calcium concentrations were taken at peak values of [Ca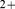]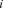, and that in following numerical experiments we performed additional initialization procedures.

**Figure 3:**
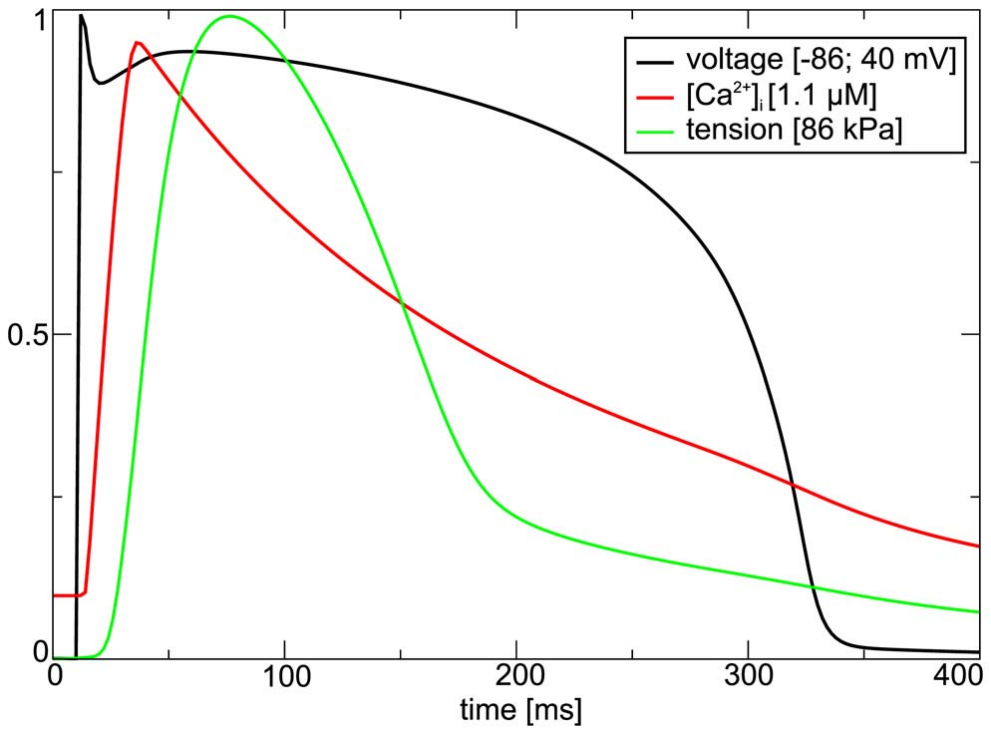
Electromechanical activity of an isolated fiber. A pulse is initialized at time 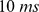 by setting voltage to 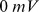 for 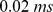. Fiber was kept at its resting length during the simulation.

Figure 3 shows an electromechanical pulse of a single fiber during isometric contraction. One can see that as in experimental records [39] the tension is slightly delayed from the [Ca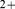]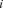 transient, the fiber produces a maximal contractile tension of 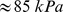 approximately after 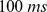 after the upstroke of the action potential.

## Results

We applied our discrete electromechanical model to study the effects of stretch-activated currents and stretch conditions on action potential duration (APD) and conduction velocity (CV) restitution, and spiral wave dynamics. The results of these studies are shown in this section.

We consider two mechanical conditions, a constantly stretched medium, and a contracting medium. The condition of sustained stretch in the medium has been assumed previously, for example, in a model to study how dilated cardiomyopathies may affect defibrillation efficacy [27], and in a model to study the effect of mechano-electrical feedback on the action potential of ventricular cells [28]. However, under normal physiological conditions cells are not constantly stretched, but contract during most of the action potential. It is interesting to note, that as an experimental condition, constantly applied mechanical load is often applied to study effects of mechano-electrical feedback, for example, in cardiac cell cultures [40] or animal models [41]. Therefore, we perform studies both, in a constantly stretched medium, and in a contracting medium to investigate the effects of different mechanical conditions.

### Action Potential Shape and Restitution Properties

#### Constant stretch

**Figure 4:**
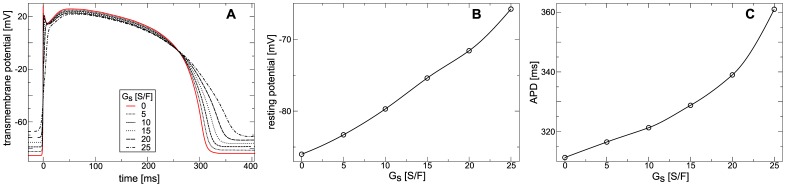
Effect of stretch-activated currents on the action potential in constantly stretched medium. (**A**) Action potential *vs* 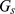. (**B**) Resting membrane potential *vs* 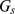. (**C**) APD *vs* 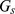. Traveling plain waves were periodically (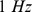) induced in a medium held at (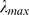) for different 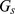. Action potentials were measured after 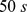. Resting potential was measured in the medium without external stimulations. APD was measured at 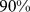 recovery.

We used a 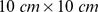 medium which we assumed stretched to 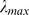. From Eq.(12) we see that in this situation every cell in the medium experiences 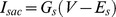, thus we apply 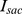 to every cell without actually deforming the medium. We initiated a train of traveling plain waves with a period of 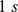 to study the influence of 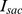 on characteristics of the action potential for 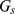 from 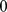 to 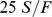. In Figure 4A we show the shape of the action potential of the cell in the center of the medium for different 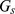 after 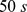 application of the stimulation protocol. From Figure 4A we see that increasing 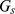 causes an increase in the resting potential in the medium and increase in APD. In particular, Figure 4B shows that increase of 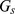 from 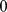 to 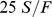 increases the resting potential by 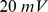 (from 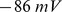 to 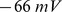). Figure 4C shows that increasing 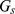 from 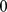 to 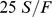 increases APD from 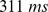 to 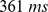. This effect occurs as a larger 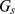 causes a stronger depolarizing 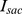. As a result of stronger 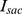 the resting potential increases, and during an action potential 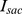 counteracts repolarizing currents elongating the APD.

**Figure 5:**
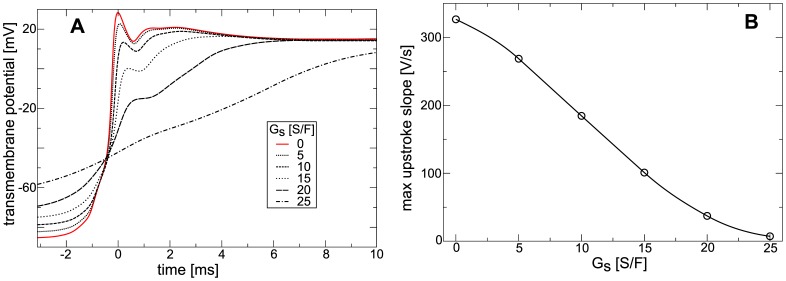
Effect of constant stretch on the action potential upstroke. (**A**) Upstroke of action potential *vs* 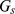. (**B**) Maximal upstroke slope *vs* 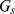. Protocol was as in Figure? 4.

The effect of 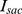 on the upstroke of the action potential in the constantly stretched medium is illustrated in Figure 5. We see in Figure 5A that the upstroke peak and slope decreases with increasing 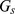, and that for 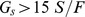 no typical sodium driven upstroke takes place. In Figure 5B the upstroke slope is shown as a function of 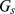. We see that for 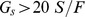 the upstroke slope drops to values under 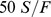 which is much lower than the sodium driven upstroke in the TP06 model without deformation. This effect of 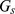 on the upstroke can be explained by a depletion of fast sodium channels via the accommodation phenomenon, a decrease of opening probability of fast sodium channels due to sub-threshold depolarization [29]. In a previous study [28], which also assumed a constantly stretched medium, similar effects of 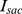 on the APD, upstroke and resting potential were found. Moreover, it was shown previously in Langendorff-perfused rabbit hearts that sustained volume load causes increased resting potential and decreased the slope of the action potential upstroke [41].

**Figure 6:**
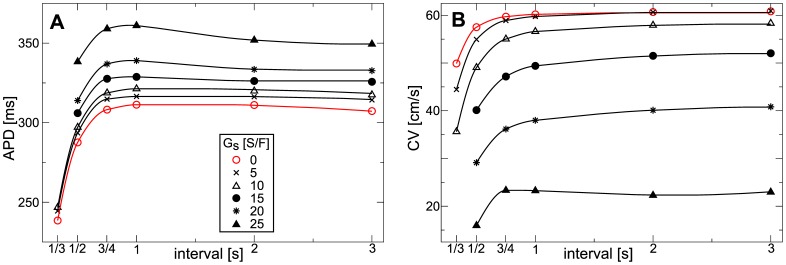
Dependence of restitution on stretch-activated currents in constantly stretched medium. (**A**) APD restitution *vs* 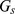. (**B**) CV restitution *vs* 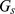. Same parameters were used as in Figure? 4.

To measure restitution properties in the constantly stretched medium we applied the same setup as above, but varied the stimulation period from 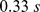 to 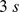. We measured CV from the difference in front arrival times between two points, one at the center, and the other 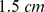 further in propagation direction. Figure 6 illustrates the APD and CV restitution. We see from Figure 6A that increasing 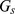 increases the APD. For 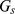 between 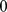 and 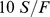 the slope of the APD restitution curve is not affected much, and APD grows continuously for longer stimulation periods to a plateau. For 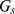 between 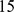 and 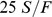 and a stimulation period longer than 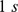 we see that an increasing, slightly negative slope of the APD restitution curve is caused. From Figure 6B we see, that increasing 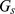 causes decreasing CV. For stimulation intervals shorter than 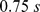 we observe that increasing 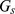 causes a higher steepness of the CV restitution curve. Restitution curves for 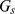 between 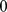 and 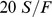 are monotonically increasing for longer stimulation intervals; yet, for 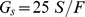 we see a biphasic shape with a local maximum of around 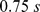, and slightly negative slope of the CV restitution curve for longer stimulation interval. We also see that the minimal period of excitation increases with increase of 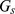 from about 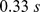 for 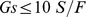 to about 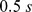 for 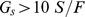. In a previous study [27], where also constant stretch was assumed in the medium, increasing 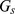 also caused longer APD and decreased CV.

#### Contracting Medium

**Figure 7:**
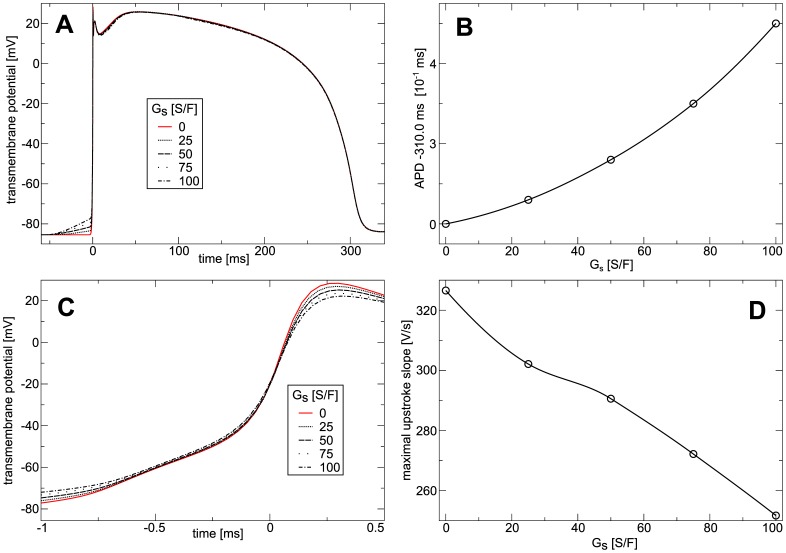
Effect of stretch-activated currents on the action potential in contracting medium. (**A**) Action potential *vs* 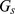. (**B**) APD *vs* 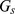. (**C**) Upstroke of action potential *vs* 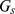. (**D**) Maximal upstroke slope *vs* 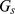. Traveling plain waves were periodically (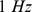) induced for different 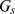. Action potentials were measured after 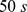. Resting potential was measured in the medium without external stimulations. APD was measured at 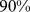 recovery.

For the simulations in a contracting medium we applied the same system size, stimulation protocol, and parameter setting as for the constantly stretched medium; however, the medium is deforming due to excitation-contraction waves, and the boundaries are fixed space (see section “Numerical Methods”). In Figure 7A we show how the shape of the action potential is affected by 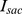. We see that increasing 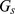 in from 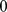 to 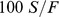 causes no substantial increase in the resting potential and in APD. Another effect of increasing 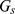 is a linear increase in the transmembrane potential starting 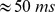 prior the upstroke. For example, for 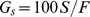 the transmembrane potential increases to 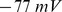, which is well under the threshold of excitation, that is at 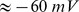. In Figure 7B we illustrate the effect of 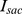 on APD. One can see that increasing 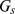 from 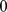 to 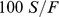 increases APD by 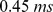. This effect of 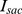 on APD is small in the studied parameter range compared to the constantly stretched medium, where increasing 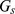 from 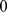 to 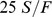 resulted in increase of APD by 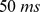.

In Figure 7C we illustrate the effect of 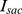 on the action potential upstroke in the deforming medium. We can see that the upstroke peak decreases for increasing 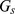; from 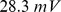 (for 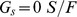) to 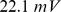 (for 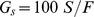). This decrease in upstroke peak is small compared to the constantly stretched medium, where an increase of 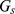 from 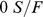 to 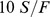 caused a decrease of the upstroke amplitude by 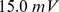 (compare Figure 5A). Furthermore, in Figure 7D we illustrate the maximal upstroke slope against 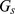. The maximal upstroke slope decreases for increasing 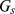. The effect of 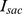 is small compared to the constantly stretched medium. In particular, increasing 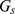 from 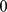 to 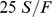 in the contracting medium decreases the upstroke slope from 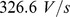 to 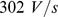, whereas in the constantly stretched medium an increase of 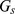 from 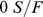 to 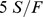 causes a decrease to 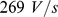.

**Figure 8:**
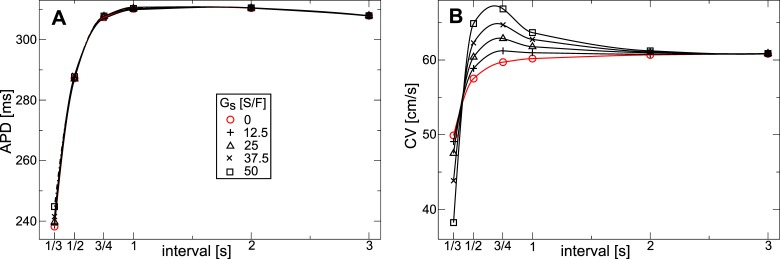
Dependence of restitution on stretch-activated currents in contracting medium. (**A**) APD restitution *vs* 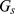. (**B**) CV restitution *vs* 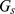. Same parameters were used as in Figure? 6.

Figure 8A illustrates the effect of 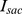 on the APD restitution in the contracting medium. From Figure 8A we see a small effect of 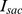 on the APD. Only for a stimulation period shorter than 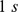 we see a small decrease in steepness of the restitution curve with increasing 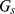.

Figure 8B illustrates the effect of 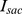 on CV restitution in the contracting medium. We see, that for a stimulation period longer than 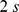 stretch activated currents have only little effect on CV. This is because for a slow stimulation period the effects of deformation caused by a preceding wave progressively decrease. For stimulation periods shorter than 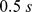, a steep positive CV restitution is present for all measured values of 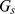, and the slope of the CV curve increases when 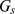 is larger. For stimulation periods between 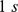 and 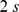 we see that contraction results in negative CV restitution slopes: a higher periodic stimulation causes higher wave velocities. Note that CV depends on the position it is measured, as the medium before the wave is depolarized by 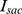. Here we used an average CV to illustrate the abnormal CV restitution.

#### Effect of Mechanical Conditions

**Figure 9:**
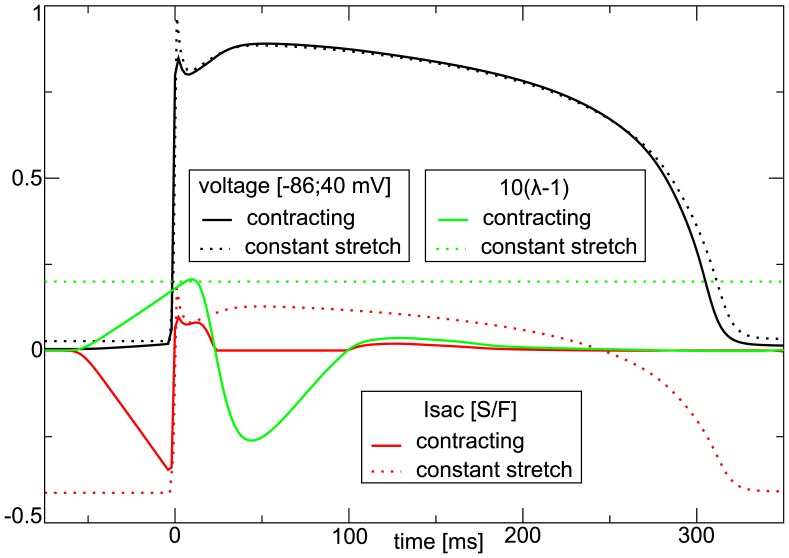
Effect of stretch conditions on stretch-activated currents and action potential shape. Continuous lines show variables for cell in the medium, dotted lines show variables for constantly stretched cell. The constantly stretched cell was constantly held at 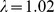, and paced at 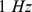 to steady state dynamics. The other cell was in a contracting medium in the same setup as in Figure? 6, and also paced at 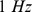 to steady state dynamics. 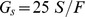.

Let us now compare the results for a constantly stretched and contracting medium. We found, that under both, dynamical and static stretch conditions, increasing 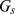 causes an elongation of the APD; however, in the deforming medium the effect is much smaller than in the constantly stretched medium (compare Figures 6A, and 4 with Figures 7B, and 8A). Furthermore, the results of the CV restitution in the constantly stretched and the contracting medium are significantly different. We can explain these differences by substantially different time courses of stretch in a constantly stretched and in contracting tissue. Figure 9 illustrates the shape of action potential, stretch activated current, and deformation of the medium for a single cell which is subject to a constant stretch (similar to Figure 4), and for a cell in a contracting medium, in which we measured the APD restitution shown in Figure 8. In both setups the cell was paced at 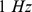. We see that 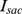 in a constantly stretched cell is active during the entire action potential, and 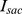 has a substantial negative value (inward current) at the waveback, which results in APD prolongation. On the contrary, for a cell in the contracting medium 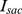 is absent at the waveback. This is because at this phase of the action potential the cell is contracting, and thus no stretch activated current is produced. As a result of that difference, the APD for a cell in the deforming tissue is only slightly longer than that of a cell in a non-deforming tissue. Some elongation of the APD at increased 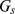 can be explained with the negative current prior/during the upstroke of the action potential which slightly decreases the sodium current via the accommodation phenomenon. The linear increase of transmembrane potential shown in Figure 7 is also present in Figure 9, and we can see that it is caused by a linear increase of stretch and thus 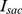 which sets in 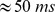 prior the upstroke. Overall, in the deforming medium the cell is affected by 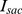 only from 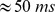 before the upstroke until 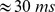 after the upstroke, while during constant stretch 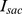 is always present. This explains why the effect of 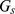 on the APD is much smaller in the contracting medium compared to the constantly stretched medium. Note the change in sign of 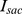 when the transmembrane potential reaches the reversal potential of the stretch activated channels 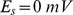 (compare Eq.(12)). Thus 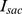 has a depolarizing effect on cardiac cells prior an action potential, and can have a repolarizing effect during the action potential. From Figure 9 we can also understand the different results on the CV restitution curves for the constantly stretched and the contracting medium. In the constantly stretched medium decrease of velocity is due to the constantly depolarizing 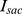 causing accommodation, whereas in the contracting medium, a depolarization of the medium 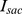 sets in only about 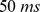 prior to the upstroke (compare Figure 4), so that no significant accommodation takes place, and causes a preexcitation of the medium prior the traveling wave. As a result, for intermediate stimulation periods, an increase in 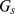 causes an increase of CV.

### Spiral Wave Dynamics

**Figure 10:**
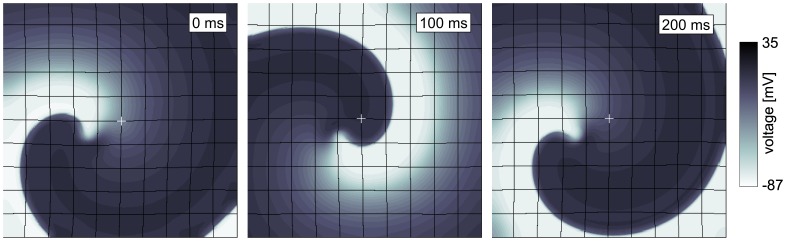
Illustration of spiral wave dynamics in contracting medium. Time after stretch activated current 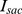 was activated is shown top right of each subfigure. 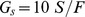.

We studied effects of deformation on spiral wave dynamics in our model. We initiated a spiral wave with an S1–S2 protocol in the medium, and then simulated spiral rotation for 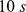 to avoid artifacts from the spiral initiation protocol. During this initial phase 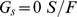. The values of all variables are then recorded, and used as initial conditions for the following simulations. We set 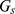 to various values, in contracting and constantly stretched medium, and studied how this affects the dynamics of the rotating spiral wave. We studied spiral wave dynamics for 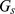 between 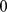 and 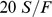. Figure 10 illustrates the simulation experiment, it shows spiral rotation in the model for 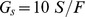 in the contracting medium.

**Figure 11:**
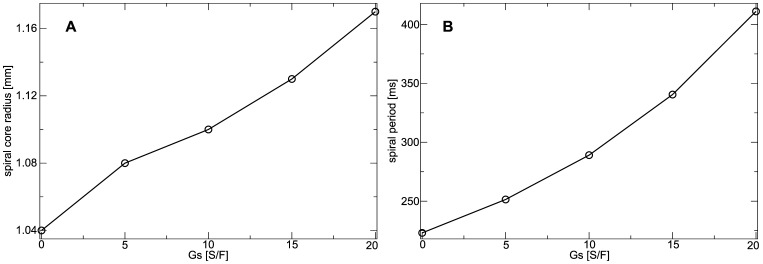
Dependence of spiral wave dynamics on stretch-activated currents in constantly stretched medium. (**B**) Spiral core radius as a function of 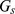. (**C**) Spiral wave period as function of 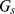.

We found that in the constantly stretched medium (assumed to be stretched to 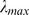) the spiral tip follows a static circular core (data not shown). Figure 11A illustrates how 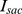 affects the spiral core radius. We found that the size of the spiral wave core increases with increasing 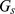 (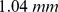 for 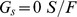, and 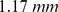 for 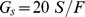). In Figure 11B it is illustrated how the spiral period is affected by 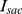 in the constantly stretched medium. We find, that the spiral period increases for increasing 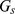. An increase of 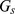 from 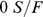 to 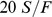 causes an increase of spiral period from 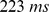 to 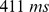. This increase in the period can be explained by elongation of APD under constant stretch conditions (Figure 9).

**Figure 12:**
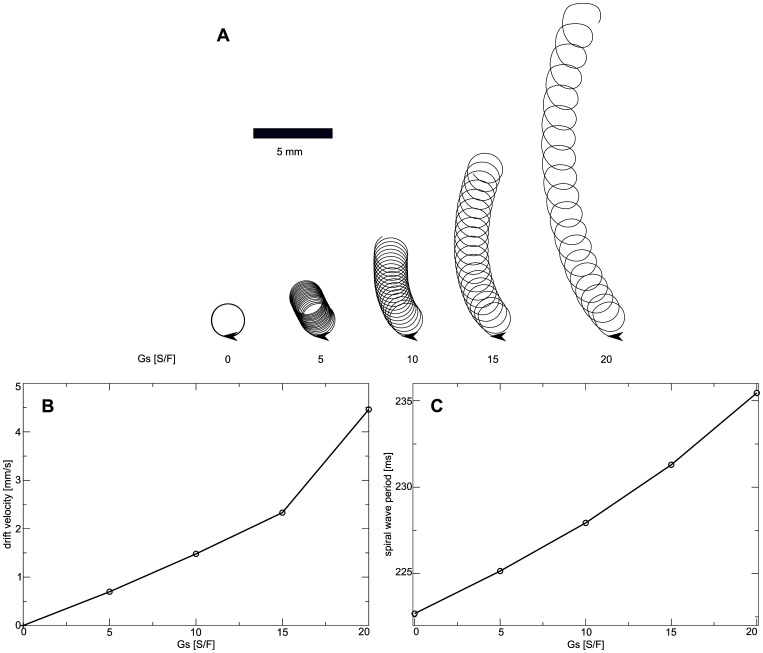
Dependence of spiral wave dynamics on stretch-activated currents in contracting medium. (**A**) Spiral tip trajectories are shown for different values of 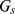. Each tip trajectory illustrates drift for 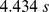. Starting points and drift directions are illustrated with arrows. (**B**) Spiral wave drift velocity as function of 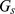. Drift velocity is estimated from the tip trajectories using the distance of the spiral core position. (**C**) Spiral wave period as function of 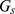. Spiral period was measured from the last spiral rotation.

Figure 12A illustrates how the spiral wave rotation is affected by 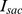 in the contracting medium. We see that in absence of 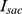 the spiral rotates around a circular core. However, for increasing 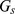 the spiral starts to drift, and drift velocity increases with an increase of 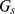. All spiral tip trajectories in Figure 12A show drift for the same time interval (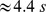), and we see that the distance traveled by the spiral tip increases substantially with an increase of 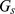. We use the traveled distance of the spiral tip to estimate the velocity of spiral wave drift. Figure 12B shows the velocity of spiral wave drift as a function of 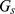. We see an approximately linear increase in drift velocity with increase of 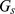. Figure 12C illustrates the effect of 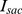 on spiral wave period. We see that increasing 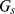 increases the spiral wave period: an increase of 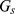 from 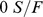 to 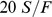 causes an increase of spiral period from 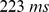 to 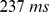. This effect can be explained by the accommodation phenomenon. 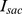 decreases the availability of sodium channels which results in a decrease of excitability which is known to increase the period of a spiral wave. However, as the effect of 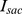 on APD is minimal (see Figure 8A) the increase of the period is also less substantial compared to the constantly stretched medium. Moreover, a decrease in excitability of a medium is known to increases the radius of a spiral core [42]. We calculated the size of the core of drifting spirals by correcting the spiral tip position data for the drift of the core, and indeed found some increase of the core radius with increasing 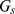 (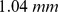 for 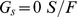, and 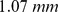 for 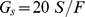); however, the effect is small.

Our results on spiral wave drift in contracting tissue are qualitatively similar to that observed in [14], where a low dimensional model of cardiac tissue was applied, and the spiral drift was discussed as a so-called resonant drift mechanism [43]. Resonant-drift can be induced by a periodical variation of the medium properties such as its excitability synchronously with the spiral wave period [44]. In our model a rotating spiral wave itself periodically affects the excitability of the medium. We can understand this from Figure 10, where we can see that the fraction of the excited surface area (and thus the fraction of contracting medium) to the total surface area of the medium changes in synchrony with the spiral rotation, in turn affecting the mediums excitability properties. Therefore, we believe that in our simulations and in [14] the underlying mechanism of spiral drift is the resonant drift.

## Discussion

We introduced a discrete electromechanical model of the human heart which couples a biophysical model of cardiac excitation [20], [21] and tension development [22], [23] with a discrete elastic mass-lattice model. We demonstrated the value of the model in an application study. We used our new model to investigate how stretch conditions and stretch-activated currents affect the heart's functioning. For this we studied how stretch-activated currents affect action potential shape, restitution properties, and spiral wave activity in a medium which we assumed constantly stretched, and a contracting medium with isometric boundary conditions. We found that stretch conditions significantly influence these properties by activating stretch-activated ion channels. In the freely deforming medium we find that the primary effects are accommodation, and preexcitation of the medium. In the constantly stretched medium we find a much stronger accommodation effect, no effect of preexcitation, and substantial elongation of the APD caused by depolarizing 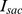 during the recovery phase of the action potential. We found that spiral wave drift is caused in the deforming medium, whereas in the constantly stretched medium rotation dynamics is not affected, but spiral period and core size is increased.

It has been shown that the dynamics of spiral waves in the heart manifests itself in the type of cardiac arrhythmia, for example, a drifting spiral wave can induce a polymorphic ventricular tachycardia which is a known precursor for ventricular fibrillation [45]. Our results show that in addition to heterogeneity induced spiral wave drift [46]–[48] there is a drift due to mechano-electrical feedback which can also affect the type of cardiac arrhythmia.

Our results on restitution properties suggest that in dynamic stretch-conditions 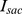 causes abnormal CV restitution due to a preexcitation in the medium. It has been shown that abnormal CV restitution can cause alternans and initiation of spiral waves [49], [50], and also important phenomena on spiral wave dynamics such as discordant alternans can be caused by abnormal CV restitution [51], [52]. We expect that this mechanism of mechanically caused abnormal CV-restitution is important to understand the onset of arrhythmias due to emergent dynamic inhomogeneities.

The computation time of our mechanical model scales linearly against the number of mechanical nodes, which allows to solve the model with a higher mechanical node density and thus high spatial resolution of 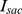 [16]. Furthermore, this computational efficiency of the discrete mechanical model allows us to update the its configuration after each electrical step (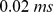). Continuous mechanical studies on cardiac function normally solve mechanics following several electrical steps, because its more demanding numerical schemes, for example in [18] the mechanical configuration was solved following 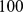 electrical steps.

The passive elasticity of the heart is most commonly described by hyperelastic constitutive relations in finite element formulations of continuum mechanics, for example, the Guccione material relation in [18]. A drawback of the mass-lattice framework of the new model is its difficulty to reproduce passive mechanical properties of biological tissue with a discrete mechanical model, for example volume conservation or specific passive mechanical properties such as hyperelasticity. In contrast, these properties can be directly described in constitutive relations in continuum mechanics. The discrete electromechanical model could be extended to describe hyperelastic material relations, for example using the approach developed by Fritz et al. in [53], where a mass-spring model is in fact adapted to a hyperelastic material relation to describe cardiac mechanics. Moreover, also volume conservation and anisotropy of heart tissue can be introduced to discrete mechanical models [54], [55].

As another next step, the discrete electromechanical model can be extended to three-dimensional simulations to study the effect of mechano-electrical feedback on the dynamics of scroll waves.

Our modeling framework can potentially be used to estimate effects of mechanical or electrical components in experimental studies of wave propagation in the heart. However, it needs to be adjusted to the specific tissue type and mechanical properties of the experimental system. It can be done by changing the parameters on our model based on direct measurements.

We set up the new electromechanical model using a standard form for the stretch-activated currents 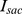 in Eq.(12). This allows us to compare the new results to results previously achieved with electromechanical models using a continuous mechanical description, for instance results on spiral wave drift in [14]. Experimental studies showed that 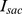 depends also on additional factors, for example, the stretch rate [56]. Our model can easily be adjusted to other formulations of 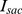, for example, to the formulation of Jie et al. [19] which considers a stretch rate dependency. It can be interesting to compare the effect of different formulations of 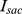 on the dynamics of wave propagation.

The effect of deformation of the medium on the metric tensor are neglected in the model, as we assume that the main resistivity between cells is constituted by gap junctions. We used this assumption also in [15], where we found that the change of the metric tensor did not affect qualitative results our study on mechanically caused pacemaking activity in a low-dimensional model.
