# Correction: A Discrete Electromechanical Model for Human Cardiac Tissue: Effects of Stretch-Activated Currents and Stretch Conditions on Restitution Properties and Spiral Wave Dynamics

**DOI:** 10.1371/annotation/9ceadf50-eb8f-4051-9e41-772884d47385

**Published:** 2013-06-24

**Authors:** Louis D. Weise, Alexander V. Panfilov

There were numerous errors in the article. Corrections to them are presented in the attached file: http://www.plosone.org/corrections/pone.0059317.001.cn.tif

